# Hepatolithiasis: A Retrospective Analysis of Surgical Management Options in a Tertiary Care Centre in Southern India

**DOI:** 10.7759/cureus.27797

**Published:** 2022-08-08

**Authors:** Sourabh Jindal, Afroj I Bagwan, Rajkumar Rathinasamy, Prabhakaran R, Sugumar Chidambaranathan, Naganath Babu O L

**Affiliations:** 1 Institute of Surgical Gastroenterology, Madras Medical College, Chennai, IND

**Keywords:** surgical management, liver abscess, hepatic resection, access loop, hepatolithiasis

## Abstract

Introduction: Hepatolithiasis (HL) is the presence of stones in the bile ducts proximal to the confluence of the hepatic ducts. This study aims to analyse the case presentations of HL in a tertiary care centre in South India and define the role of hepatic resection in these cases and their outcomes.

Methods: Retrospective data of all patients operated on for HL from 2012 to 2021 were analysed with regard to clinical parameters, biochemical parameters, and different types of surgical management. Descriptive data analyses were done.

Results: A total of 42 patients underwent surgical treatment for HL between 2012 and 2021 in our institution. Of the patients, 64% were females. A total of 50% of patients were affected by bilateral HL. Of the patients, 95% had abdominal pain, 57% had a fever, and 29% presented with jaundice. A total of 38% of patients had a history of previous biliary surgery. Atrophy was present in 38% of cases. Choledochoduodenostomy was performed in 26%, and hepatic resection with bilio-enteric anastomosis was done in 36% of patients. Endoluminal access loop was done in 21%. Hepaticojejunostomy alone was done in 14%. On follow-up visits (mean: 61.5 months), 60% of patients were asymptomatic with no recurrence. There was nil 30-day postoperative mortality.

Conclusion: The treatment options for HL were based on the extent of liver involvement. The bilio-enteric anastomosis was done after the clearance of stones for uncomplicated HL. Complicated HL may need liver resection with hepaticojejunostomy, with an endoluminal access loop for a good outcome.

## Introduction

Hepatolithiasis (HL) is defined by stones present proximal to the junction of the right and left hepatic ducts causing complete or intermittent biliary obstruction, resulting in recurrent attacks of abdominal pain, fever, and jaundice (Charcot's triad) [1].

HL is endemic in East Asia, where prevalence can range up to 50% [2]. Intrahepatic stones are uncommon in western countries, with an incidence of about 1% [3,4]; however, the incidence is steadily increasing, owing to increasing immigration [5]. HL typically develops between the ages of 30 and 70 years, with the highest incidence in the fifth and sixth decades of life [6]. Incidence is equal between both sexes. It does, however, differ greatly by country, with differences even within subpopulations [1].

The cause of HL is unknown, but ethnic factors, poor nutrition, bacterial infection, parasite infestation, and bile duct anatomy anomalies have all been suggested. Chronic parasitic infestations, particularly *Clonorchis sinensis* as a result of raw fish consumption and *Ascaris lumbricoides*, are more prevalent in Southeast Asian countries and detected in up to 30% of patients with HL [7-9].

HL is also known as "Oriental cholangiohepatitis" or "pyogenic cholangitis", and is associated with a high rate of treatment failure and recurrence [10].

Dong et al. described a classification of HL for use in determining surgical approaches. Type 1 is a localized disease and type 2 contains diffusely distributed multiple HL and is divided into three different subcategories. The presence of extra-hepatic stones in this classification is defined as type E with three subgroups [11].

Complications of HL include suppurative cholangitis, liver abscess, and chronic disease leading to biliary cirrhosis and cholangiocarcinoma; early treatment may prevent additional liver damage and improve long-term prognosis [10].

Treatment seeks to cure continuing infections, avoid recurring cholangitis and consequent hepatic fibrosis, minimize the need for repeated instrumentation, and prevent the emergence of cholangiocarcinoma, regardless of the source [11].

According to several studies, the following are the indications for hepatectomy as a therapy for HL: (i) unilobar HL, (ii) atrophy of the affected liver segments or lobe, (iii) presence of a liver abscess, (iv) cholangiocarcinoma, and (v) multiple intrahepatic stones causing marked proximal biliary stricture or dilation [12-16].

Access to the biliary tree is critical in the treatment of HL to treat residual and recurring stones as well as ductal strictures using endoscopic or percutaneous procedures. Stone retrieval through the T-tube tract or by an access loop implanted as a stoma [17] or in the subparietal layer [18] is addressed via puncture under fluoroscopic guidance. Alternatively, the Roux limb of the hepaticojejunostomy (HJ) can be anastomosed to the stomach [19] or duodenum [20] for future endoscopic management.

This study aims to evaluate the demographic and clinicopathological factors, appraise the role of surgical management, and analyse the postoperative outcomes.

## Materials and methods

This retrospective study was conducted at a high-volume tertiary care centre in Chennai. All patients with HL who were operated on in our institute during the period between 2012 and 2021 were included in the study. Data were analysed from a prospectively maintained institutional database and 42 patients were analysed in this study.

Patients were evaluated for features of cholangitis on admissions like abdominal pain, fever, and jaundice. History of previous investigations and treatment history, including endoscopic and surgical interventions, were reviewed. Complete blood count, liver function tests, and renal function tests were done in all patients. Blood culture was done as necessary. Patients were evaluated initially using ultrasound and computed tomography (CT). Magnetic resonance imaging (MRI) with magnetic resonance cholangiopancreatography (MRCP) was done to determine the location of stones, strictures, and the presence of lobar atrophy or liver abscess, and treatment was planned accordingly.

Based on the presence of stones and segmental liver atrophy, patients were classified into 1a (right HL), 1b (left HL), and 1c (bilateral HL), and surgical management options were chosen accordingly. Postoperative complications were recorded.

## Results

A total of 42 patients underwent various procedures in our institution from 2012 to 2021. Demographic and clinical characteristics are shown in Table 1. The disease was more prevalent in females with a female-to-male ratio of 1.8. The mean age was 42 years. The main symptoms were abdominal pain, fever, and jaundice. Four (9.5%) patients had a liver abscess. A total of 21 (50%) patients had bilateral HL, 16 (38.1%) had left HL, and five (11.9%) had right HL. Liver atrophy was seen in 16 (38.1%) cases. Nine (21.4%) cases showed segment 2 and 3 atrophy, and three (7.1%) cases showed segment 2, 3, and 4 atrophy.

**Table 1:** Clinical profile of 42 patients HL: hepatolithiasis.

Parameter	N (Percentage)
Age (range)	41.85 ± 11.89 years (15-72)
Sex	
Male	15 (35.7)
Female	27 (64.3)
Symptoms	
Abdominal pain	40 (95.2)
Fever	24 (57.1)
Jaundice	12 (28.6)
Charcot triad	9 (21.4)
Location of calculi	
Right HL	5 (11.9)
Left HL	16 (38.1)
Bilateral HL	21 (50)
Previous biliary surgery	16 (38.1)

A total of 26 patients (61.9%) had no history of previous biliary surgery. Among the 16 (38.1%) patients who had a history of previous surgery, cholecystectomy alone was done in seven (16.7%) patients, cholecystectomy with common bile duct (CBD) exploration and T-tube drainage was done in three (7.1%) patients, cholecystectomy with choledochoduodenostomy (CDD) was done in two (4.8%) patients, one (2.4%) patient had choledochal cyst excision, and two (4.9%) patients underwent right hepatectomy with HJ for Caroli’s disease and HL. One patient (2.4%) had a history of cholecystectomy at seven years of age and HJ at 22 years for HL. Strictures were present in eight (19%) cases. Nine patients (21.4%) had comorbid illnesses such as hypertension, diabetes mellitus, and hypothyroidism.

Table 2 shows the preoperative blood profile of the patients, with a total blood count of 9401.42 ± 3941.02. Other blood parameters are shown below.

**Table 2:** Preoperative blood profile of the patients

Parameters	Median	Range
Total leucocyte count, /mm^3^	8550	5200-24500
Total bilirubin, mg/dl	0.7	0.4-10.6
Direct bilirubin, mg/dl	0.5	0.1-10
Alkaline phosphatase, U/L	101	13-665
Total protein, g/dl	6.7	5.2-8.3
Albumin, g/dl	3.6	2.8-3.7

All the patients who had no prior biliary surgery underwent cholecystectomy. Additional surgical procedures performed were as follows: bile duct exploration with CDD alone in 11 cases (26.2%), hepatic resection with CDD in 11 cases (26.2%), HJ alone in six (14.3%) cases, hepatic resection with HJ in three (7.14%) cases, and HJ with endoluminal access loop in seven (16.67%) cases. Two (4.77%) patients underwent hepatic resection with HJ and endoluminal access loop. Eight patients underwent duodenal access loop and one underwent gastric access loop. One patient underwent right hepatectomy alone and another underwent intrahepatic cholangiojejunostomy (Longmire procedure). One patient who had liver resection was found to have incidental cholangiocarcinoma in final histopathology. Surgical procedures done in the present study are tabulated in Table 3, according to the Dong’s classification [11].

**Table 3:** Surgeries done in our series tabulated according to the Dong’s classification* CDD: choledochoduodenostomy; HJ: hepaticojejunostomy. * Adapted from [11].

Type	Definition or content	No	Type of surgery
Type I	Localized stones: unilobar or bilobar	15	CDD/HJ
Type II	Diffusely distributed stones		
IIa	Without hepatic atrophy; no stricture of the intrahepatic bile ducts	11	CDD/HJ with endoluminal access loop
IIb	Atrophy limited in segment or/and stricture of the intrahepatic bile ducts	16	Hepatic resection ± CDD/HJ with endoluminal access loop
IIC	Biliary cirrhosis and portal hypertension	Nil	Nil
Additional type E	Extrahepatic stones	18	CDD/HJ

Postoperative stone analysis showed that 28 (66.7%) patients had calcium bilirubinate stones, eight (19%) had cholesterol stones, and six (14.3%) had mixed stones. A total of 27 (64.3%) patients had no wound infection. A total of 33 (78.6%) patients showed no growth on bile culture, eight (19%) showed *Klebsiella pneumoniae*, and one (2.4%) showed *Escherichia coli*.

The mean duration of hospital stay was 34.68 ± 13.45 days, and the mean duration of postoperative stay was 13.5 ± 6.8 days. Pulmonary complications developed in two patients. Postoperatively, bile leak and bleeding developed in one patient each, and they underwent re-exploration.

The follow-up period ranged from six to 102 months with a mean follow-up of 61.5 months. A total of 25 (59.52%) patients had no recurrence. Recurrent HL developed in nine (21.42%) patients; reoperation was done in three (7.1%), endoscopic therapy was done in three (7.1%), percutaneous transhepatic cholangioscopic lithotomy in one, and another two patients were taking herbal medications, respectively. Five patients were lost to follow-up. The remaining three patients died during follow-up due to recurrent cholangitis, metastatic cholangiocarcinoma within two years of treatment, and liver cirrhosis after five years.

## Discussion

HL is an uncommon illness in western countries, and endemic in East Asian countries [11]. As the incidences are increasing, there is no clear consensus regarding the most effective treatment. In the present study, it was noted that 21 (50%) cases had bilateral HL and 16 (38.1%) had left HL, which is contrary to most of the studies, i.e., Mohanraj et al. [21] and Chen et al. [14]. In Mohanraj et al.'s study, eight out of 14 patients had left HL, and only two cases had bilateral HL. Chen et al.'s study showed that 77% of patients had stones confined to the left lobe of the liver and 14% had stones in both lobes of the liver.

In the present study, 40 (95.2%) patients had pain, and only 24 (57.1%) had a fever, which is similar to most studies where almost all the patients presented with pain and fever with a mean duration of four years [10,13,21].

In the present study, 28 patients (66.7%) had calcium bilirubinate stones, eight (19%) had cholesterol stones, and six (14.2%) had mixed stones. This is in line with Mohanraj et al.'s study, in which most of the patients had calcium bilirubinate stones. However, most western patients had cholesterol stones. This could be due to the change in the diet pattern. Asian people take a diet that is rich in carbohydrates and low in protein and fat. Low-fat diet results in decreased release of cholecystokinin, which causes biliary stasis. In contrast, low protein results in a low level of glucaro-1,4-lactone, an inhibitor of β-glucuronidase, enhancing the deconjugation process [21].

The major goals of treating HL are to remove all stones and eliminate bile stasis in the biliary system to prevent recurrence and infection. As a result, the management must be tailored to the disease's various presentations. Surgical treatment options include biliary decompression with bilio-enteric anastomosis with/without hepatic resection. Over the last few decades, both the techniques of liver resection and perioperative management have significantly improved, resulting in a remarkable decline in the morbidity and mortality of hepatic surgery [14].

Hepatectomy is the best approach for treating HL because it removes both stones and strictured bile duct, and resects the atrophic portion of the liver, thus reducing the risk of recurrent stones and eliminating the potential presence of cholangiocarcinoma [13]. Hepatic resection was combined with CDD in the present study among 11 (26.2%) cases, also combined with HJ in three (7.14%) cases. Hepatic resection was performed on those cases that showed evidence of liver atrophy on imaging. There was no in-hospital mortality and a low morbidity rate (9.5%). All four patients who had previously undergone HJ underwent anastomosis revision.

The stone recurrence rate after the complete stone removal was 21.42% in our series. Two patients who had recurrent stones switched to herbal medication.

Despite best efforts, none of the existing therapeutic approaches can guarantee total clearance, and post-treatment follow-up is necessary to deal with residual or recurring stones. Recurrent stones and cholangitis occur with a frequency of 9.5-16% following surgery and 31-40% after percutaneous or endoscopic lithotomy [22]. Despite developments in different methods of treatment for hepatolithiasis, important challenges to success include intrahepatic biliary strictures, impacted calculi, and unreachable peripheral calculi [23].

Advantages of doing CDD or access loop formation allow future endoscopic access to manage recurrent or residual stones without the need for surgery [17-20,24]. Kassem et al. [25] reported successful treatment of remnant stones and recurrent stones in their series of 42 permanent access cases (hepaticocutaneous jejunostomy).

## Conclusions

HL is a disease with varied presentations and a high risk of recurrence. Management is undertaken based on the case presentation and level of liver involvement. CDD and HJ are the most commonly employed bilio-enteric drainage procedures. Hepatic resection should be performed when there is underlying liver atrophy. Access loops are beneficial as they provide access for further reintervention.

## References

[1] Lorio E, Patel P, Rosenkranz L, Patel S, Sayana H (2020). Management of hepatolithiasis: review of the literature. Curr Gastroenterol Rep.

[2] Catena M, Aldrighetti L, Finazzi R, Arzu G, Arru M, Pulitanò C, Ferla G (2006). Treatment of non-endemic hepatolithiasis in a Western country. The role of hepatic resection. Ann R Coll Surg Engl.

[3] Shoda J, Tanaka N, Osuga T (2003). Hepatolithiasis—epidemiology and pathogenesis update. Front Biosci.

[4] Pausawasdi A, Watanapa P (1997). Hepatolithiasis: epidemiology and classification. Hepatogastroenterology.

[5] Li C, Wen T (2017). Surgical management of hepatolithiasis: a minireview. Intractable Rare Dis Res.

[6] Freise J, Mena J, Wen KW, Stoller M, Ho S, Corvera C (2020). A rare presentation of hepatolithiasis in an adolescent patient: a case report. Int J Surg Case Rep.

[7] Tazuma S (2006). Gallstone disease: epidemiology, pathogenesis, and classification of biliary stones (common bile duct and intrahepatic). Best Pract Res Clin Gastroenterol.

[8] Leung JW, Yu AS (1997). Hepatolithiasis and biliary parasites. Baillieres Clin Gastroenterol.

[9] Kim HJ, Kim JS, Joo MK (2015). Hepatolithiasis and intrahepatic cholangiocarcinoma: a review. World J Gastroenterol.

[10] Huang MH, Chen CH, Yang JC (2003). Long-term outcome of percutaneous transhepatic cholangioscopic lithotomy for hepatolithiasis. Am J Gastroenterol.

[11] Feng X, Zheng S, Xia F, Ma K, Wang S, Bie P, Dong J (2012). Classification and management of hepatolithiasis: a high-volume, single-center's experience. Intractable Rare Dis Res.

[12] Uchiyama K, Onishi H, Tani M, Kinoshita H, Ueno M, Yamaue H (2002). Indication and procedure for treatment of hepatolithiasis. Arch Surg.

[13] Chijiiwa K, Yamashita H, Yoshida J, Kuroki S, Tanaka M (1995). Current management and long-term prognosis of hepatolithiasis. Arch Surg.

[14] Chen DW, Tung-Ping Poon R, Liu CL, Fan ST, Wong J (2004). Immediate and long-term outcomes of hepatectomy for hepatolithiasis. Surgery.

[15] Sakpal SV, Babel N, Chamberlain RS (2009). Surgical management of hepatolithiasis. HPB (Oxford).

[16] Tabrizian P, Jibara G, Shrager B, Schwartz ME, Roayaie S (2012). Hepatic resection for primary hepatolithiasis: a single-center western experience. J Am Coll Surg.

[17] Barker EM, Winkler M (1984). Permanent-access hepaticojejunostomy. Br J Surg.

[18] Beckingham IJ, Krige JE, Beningfield SJ, Bornman PC, Terblanche J (1998). Subparietal hepaticojejunal access loop for the long-term management of intrahepatic stones. Br J Surg.

[19] Sitaram V, Perakath B, Chacko A, Ramakrishna BS, Kurian G, Khanduri P (1998). Gastric access loop in hepaticojejunostomy. Br J Surg.

[20] Jameel ARA, Pitchaimuthu A, Raju P, Shanmugasundaram R, Obla NB, Gounder KD (2018). Hepatico-jejuno-duodenal access loop - a modified biliary reconstruction technique for facilitated endoscopic access to biliary tree following surgery for hepatolithiasis. Int J Hepatobiliary Pancreat Dis.

[21] Mohanraj K, Joeimon JL, Aravind A, Solomon R, Balamurali R, Ramkumar G, Muthukumuran K (2017). Hepatolithiasis in south Indian scenario- a case series. IOSR J Dent Med Sci.

[22] Otani K, Shimizu S, Chijiiwa K (1999). Comparison of treatments for hepatolithiasis: hepatic resection versus cholangioscopic lithotomy. J Am Coll Surg.

[23] Wen XD, Wang T, Huang Z, Zhang HJ, Zhang BY, Tang LJ, Liu WH (2017). Step-by-step strategy in the management of residual hepatolithiasis using post-operative cholangioscopy. Therap Adv Gastroenterol.

[24] Hamad MA, El-Amin H (2012). Bilio-entero-gastrostomy: prospective assessment of a modified biliary reconstruction with facilitated future endoscopic access. BMC Surg.

[25] Kassem MI, Sorour MA, Ghazal AH, El-Haddad HM, El-Riwini MT, El-Bahrawy HA (2014). Management of intrahepatic stones: the role of subcutaneous hepaticojejunal access loop. A prospective cohort study. Int J Surg.

